# The anti-inflammation and skin-moisturizing effects of *Boehmeria tricuspis*-mediated biosynthesized gold nanoparticles in human keratinocytes

**DOI:** 10.3389/fphar.2023.1258057

**Published:** 2023-10-06

**Authors:** Thi Hoa My Tran, Rongbo Wang, Hoon Kim, Yeon-Ju Kim

**Affiliations:** ^1^ Graduate School of Biotechnology and College of Life Science, Kyung Hee University, Yongin, Republic of Korea; ^2^ Department of Food and Nutrition, Chung Ang University, Anseong, Republic of Korea

**Keywords:** *Boehmeria tricuspis*, gold nanoparticles, inflammatory skin diseases, hyaluronic acid, HaCaT keratinocytes

## Abstract

**Introduction:** Recently, nanotechnology has emerged as a potential technique for skin generation, which has several treatment advantages, such as decreased drug cytotoxicity and enhanced skin penetration. *Boehmeria tricuspis* (BT) belongs to the *Urticaceae* family and is rich in phenolic and flavonoid compounds. In this study, we biosynthesized gold nanoparticles (BT-AuNPs) using BT extract to explore their anti-inflammatory and skin-moisturizing properties in keratinocytes.

**Methods:** Field-emission transmission electron microscopy, energydispersive X-ray spectrometry, dynamic light scattering, and Fourier-transforminfrared spectroscopy were used to examine the synthesized BT-AuNPs. qRT-PCR, western blot, and ELISA were applied for investigating the effect of BT-AuNPs on anti-inflammation and moisturizing activity in HaCaT cells.

**Results:** At concentrations below 200 μg/mL, BT-AuNPs had no cytotoxic effect on keratinocytes. BT-AuNPs dramatically alleviated the expression and secretion of inflammatory chemokines/cytokine, such as *IL-6*, *IL-8*, *TARC*, *CTACK*, and *RANTES* in keratinocytes stimulated by tumor necrosis factor-*α*/interferon-*γ* (T + I). These anti-inflammatory properties of BT-AuNPs were regulated by inhibiting the NF-κB and MAPKs signaling pathways. Furthermore, BT-AuNPs greatly promoted hyaluronic acid (HA) production by enhancing the expression of hyaluronic acid synthase genes (*HAS1*, *HAS2*, and *HAS3*) and suppressing the expression of hyaluronidase genes (*HYAL1* and *HYAL2*) in HaCaT cells.

**Discussion:** These results suggest that BT-AuNPs can be used as a promising therapeutic alternative for treating skin inflammation. Our findings provide a potential platform for the use of BT-AuNPs as candidates for treating inflammatory skin diseases and promoting skin health.

## Graphical Abstract

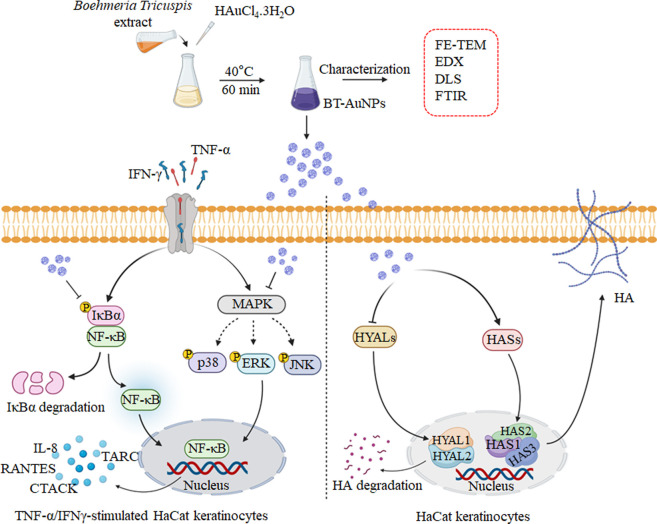

## 1 Introduction

The skin plays a critical role in the immune system, protecting against external stimuli while maintaining tissue homeostasis. However, an uncontrolled or abnormal immune response in the skin can lead to inflammatory skin diseases (ISDs), such as contact, atopic, seborrheic, and allergic dermatitis (Wang et al., 2022a). ISDs are associated with the overexpression of pro-inflammatory mediators, such as cytokines and chemokines (Wang et al., 2022a). In particular, keratinocytes might facilitate or enhance the inflammatory response by increasing the production of *TNF-*α and *IFN-γ*, leading to various skin diseases (Lin et al., 2018). In response to pro-inflammatory stimuli, keratinocytes naturally produce a distinct set of chemokines and cytokines including IL-6, IL-8, thymus and activation-regulated chemokine (TARC), and Regulated upon Activation, Normal T Cell Expressed and Presumably Secreted (RANTES) (Yang et al., 2015). These factors stimulate inflammatory cells and their infiltration into inflammatory skin lesions (Nedoszytko et al., 2014). Therefore, reducing inflammatory chemokine and cytokine secretion in keratinocytes could be an effective treatment strategy for ISDs (Kwon et al., 2012).

Recently, there has been a significant interest in nanotechnology due to the potential medical, pharmacological, and technological applications of metal nanoparticles, which have high biocompatibility (Moldovan et al., 2017). Gold nanoparticles (AuNPs) have been widely studied in biomedicine for their anti-inflammatory and antioxidant effects and their potential for drug delivery (Yamada et al., 2015). However, the conventional method for synthesizing AuNPs has several drawbacks including their hazardous natures and higher cost (Wallyn et al., 2019). To address these issues, researchers have utilized biological systems, such as microbial and plant-derived products for nanoparticles synthesis, which offer a safer and more eco-friendly approach (Mohanpuria et al., 2008). Recently, the green synthesis of nanoparticles (NPs), which involves the use of natural plant products, is becoming increasingly popular due to its nontoxic and eco-friendly approach for developing high-energy efficient materials (Hutchison, 2008). Therefore, utilizing natural plants to synthesis AuNPs may be a potential strategy to achieve results with enhanced safety and effectiveness.

*Boehmeria tricuspis* (BT) belongs to the *Urticaceae* family and is naturally grown in China, Japan, and Korea (Tang et al., 2016). The BT extract is rich in phenolic and flavoloids content which associated to significant anti-inflammatory and antioxidant activities (Chen et al., 2014; Akter et al., 2018). In addition, Chen et al. investigated the pharmaceutical application of BT leaves as potential anticancer agents (Chen et al., 2014). However, to our knowledge, the anti-inflammatory activity of AuNPs prepared with BT extract has not been investigated. Thus, this study aimed to biosynthesize AuNPs using BT extract to investigate the anti-inflammatory and moisturizing effects of BT-AuNPs on skin cells. Furthermore, we investigated the mechanisms underlying the anti-inflammatory activity of the AuNPs, particularly their involvement in NF-κB/MAPK pathway.

## 2 Material and methods

### 2.1 Materials

Dulbecco’s modified Eagle’s medium (DMEM), penicillin/streptomycin (P/S), and fetal bovine serum (FBS) were obtained from GenDEPOT (San Antonio, TX, United States). MTT (2,2-diphenyl-1-picrylhydrazyl) was provided by Thermo Fisher Scientific (Waltham, MA, United States). Primers for *IL-6*, *IL-8*, *TARC*, *CTACK* and *RANTES* were designed by Macrogen (Seoul, Republic of Korea) and their sequences are summarized in Supplementary Table S1. β-actin was purchased from Santa Cruz Biotechnology (Dallas, TX, United States) and other antibodies against p-IκBα, p-p38, P38, p-ERK 1/2, ERK 1/2, JNK, p-JNK, NF-κB, IκBα, and p-NF-κB were provided by Cell Signaling Technology (CST, MA, United States).

### 2.2 Methods

#### 2.2.1 Preparation of *B. tricuspis* extract

The leaf tissue of BT was collected from northern Gyeonggi, South Korea, located near the demilitarized zone. The plant species were identified by Dr. J. K. Kim, a senior researcher at the Gyeonggido Business and Science Accelerator, Gyeonggi Biocenter (Suwon, South Korea), and the voucher specimen was GB-0011. The leaf tissues were then sun-dried. All dried tissues were extracted for 3 days in 70% ethanol at room temperature. The extract was filtered using a filtering cloth (20 nm; Hyundai Micro, Anseong, South Korea) and vacuum-evaporated (Buchi Korea Inc., Gwangmyeong, South Korea).

#### 2.2.2 Biosynthesis and optimization of BT-AuNPs

BT-AuNPs were biologically synthesized and optimized according to a previous publication, with slight modifications (Tran et al., 2022). The following four parameters were considered during the biosynthesis optimization process: BT concentration (range: 0.25–2 mg), tetrachloroauric(III) acid trihydrate (HAuCl_4_⋅3H_2_O) concentration (range: 0.5–2 mM), reaction temperature (20°C–50°C), and incubation time (range: 20–50 min). To synthesize BT-AuNPs, a solution of 1 mL BT was mixed with a solution of HAuCl_4_⋅3H_2_O at the indicated concentrations. The reaction mixture was centrifuged at 13,500 rpm for 20 min to harvest the AuNPs. The resulting pellets were washed thrice with distilled water and dried at room temperature.

#### 2.2.3 Characterization of BT-AuNPs

The BT-AuNPs were characterized using various techniques, according to a previous report (Liu et al., 2020). The absorbance spectra of the BT-AuNPs solution were measured using ultraviolet-visible (UV-Vis) spectroscopy, while the morphology and size of the particles were analyzed using FE-TEM and DLS (Shikha et al., 2020).

Specifically, the absorbance spectra of the purified nanoparticle suspension were verified using a UV-Vis spectrophotometer (Cary 60; Agilent, Santa Clara, CA, United States). The morphology, structure, purity, and elemental distribution of BT-AuNPs were evaluated using FE-TEM. The sample was prepared by placing droplets of purified nanoparticles dispersed in water on a carbon-coated copper grid and dried at 37°C before being transferred to the microscope. Additionally, the DLS (Otsuka Electronics, Shiga, Japan) were used to measure volume, number and intensity distribution the nanoparticles size. Spectrum™ One FTIR Spectrometer (PerkinElmer, Waltham, Massachusetts, United States) were used to acquire the FTIR spectra of dried BT-AuNPs and BT. FTIR analysis was employed to determine the functional groups of the plant extract which capped on the AuNPs surfaces.

#### 2.2.4 Cell culture and cytotoxicity evaluation

HaCaT cells (Heidelberg, Germany) were cultured in DMEM supplemented with 10% FBS and 1% P/S at 37°C with 5% CO_2_/95% air. To assess the cytotoxic effects of BT-AuNPs and BT, cells were individually seeded into a 96-well plate (1 × 10^6^ cells/mL; SPL, Pocheon, Republic of Korea) for 24 h. Thereafter, various doses of BT-AuNPs and BT samples dissolved in free DMEM were added to the cells. The cytotoxic effects of the BT-AuNPs and BT samples were measured using the MTT assay.

#### 2.2.5 Live/dead fluorescent assay

Live/dead staining was performed to evaluate the cytotoxicity of BT-AuNPs and BT on HaCaT cells. Briefly, HaCaT cells were incubated with a live/dead assay kit (Thermo Fisher Scientific) for 30 min at 37°C in the dark. Cells were evaluated using a fluorescence microscope (Leica, Wetzlar, Germany).

#### 2.2.6 Analysis of cytokine, chemokine and hyaluronic acid production

After treating the samples for 24 h, the supernatant was collected from the treatment media to measure the secretion of *IL-6*, *IL-8*, *TARC/CCL17*, and hyaluronic acid (HA) using ELISA, according to the manufacturer’s instructions (R&D Systems, Minneapolis, MN, United States).

#### 2.2.7 Quantitative reverse transcription-polymerase chain reaction

HaCaT cells (1 × 10^6^ cells/mL) were seeded in a 35 × 10 mm cell culture dish for 24 h. The cells were then treated with various concentrations of BT-AuNPs for an additional 24 h. Total RNA was extracted and then cDNA was synthesized using a cDNA synthesis kit (NanoHelix). qRT-PCR was performed using the RealHelix™ Premier qPCR Kit (NanoHelix).

#### 2.2.8 Western blotting

Similarly, HaCaT cell culture and sample treatment were performed for Western blot analysis. Western blotting was performed according to a previously reported method (Mi et al., 2021). The protein blots were exposed using ATTO LuminoGraph III Lite machine (Atto, Tokyo, Japan). The ImageJ software was used to determine the intensity of the visualized bands.

#### 2.2.9 Statistical analysis

All experiments were performed in triplicate, and the data are displayed as the mean ± standard deviation. Student's t-test was used to statistically compare the two groups. Statistical significance was determined at *p* < 0.05, *p* < 0.01, and *p* < 0.001.

## 3 Results

### 3.1 Synthesis and optimization BT-AuNPs

**FIGURE 1:**
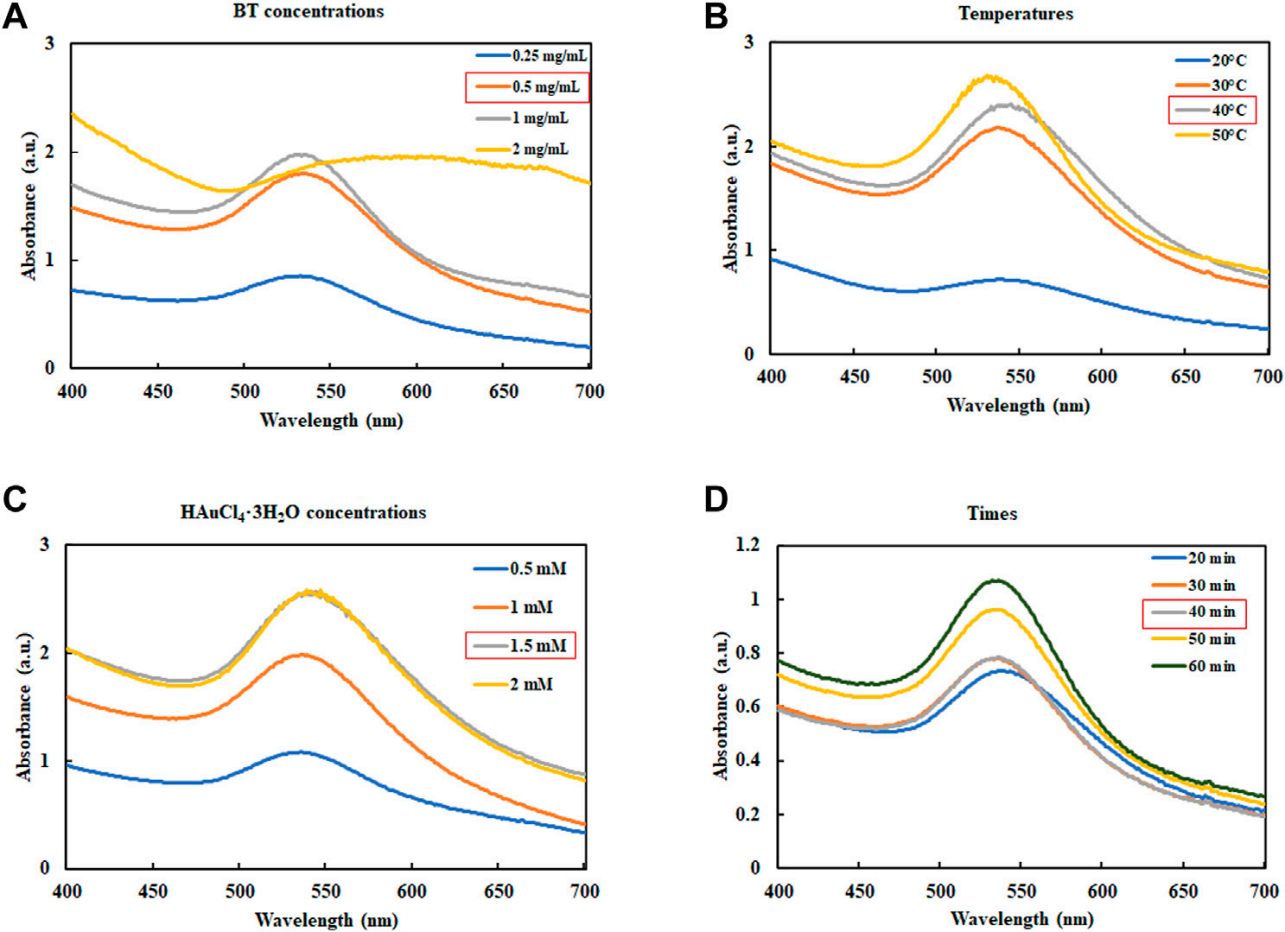
Optimization reaction condition of BT-AuNPs. **(A)** The concentrations of BT extract (BT) range from 0.25 to 2 mg/mL; **(B)** The consequence of reaction temperature (20°C–50°C); **(C)** The influence of HAuCl_4_⋅3H_2_O concentrations (0.5–2 mM); and **(D)** The consequence reaction time (20°C–50°C).

The synthesis conditions were optimized according to a previous report describing the synthesis of AuNPs using plant extracts (Elbagory et al., 2019). The formation of AuNPs was verified using UV-Vis spectroscopy. Figure 1 illustrates the UV-Vis spectra used to confirm the formation of AuNPs under various conditions. Surface plasmon wavelengths of AuNPs were analyzed at various concentrations of BT-AuNPs (0.25, 0.5, 1, and 2 mg/mL) and 1 mM HAuCl_4_⋅3H_2_O reacted at room temperature (25°C), with a total reaction time of 30 min (Figure 1A). Optimal BT-AuNP production was observed at 0.5 and 1 mg/mL; however, 0.5 mg/mL (lower concentration) was chosen for further experiments since its use was more appropriate and economical in biological applications. Subsequently, 0.5 mg/mL BT and 1 mM HAuCl_4_⋅3H_2_O were incubated at various temperatures (20, 30, 40, and 50°C) for 30 min (Figure 1B), and the optimal temperature for the production of BT-AuNPs was found to be 40°C. Further, various concentrations of HAuCl_4_⋅3H_2_O (0.5, 1, 1.5, and 2 mM) were reacted with BT (0.5 mg/mL and 40°C) for 30 min (Figure 1C). The absorbance peak was more significant at the 1.5 mM concentration. Finally, BT (0.5 mg/mL) and HAuCl_4_⋅3H_2_O (1.5 mM) were used to synthesize BT-AuNPs for different durations (20, 30, 40, and 60 min) at 40°C. The major absorption peaks were more significant at 40 min (Figure 1D). In summary, the biosynthesis of BT-AuNPs was optimal when BT (0.5 mg/mL) reacted with HAuCl_4_⋅3H_2_O (1.5 mM) at 40°C for 40 min.

### 3.2 Characterization of BT-AuNPs

**FIGURE 2:**
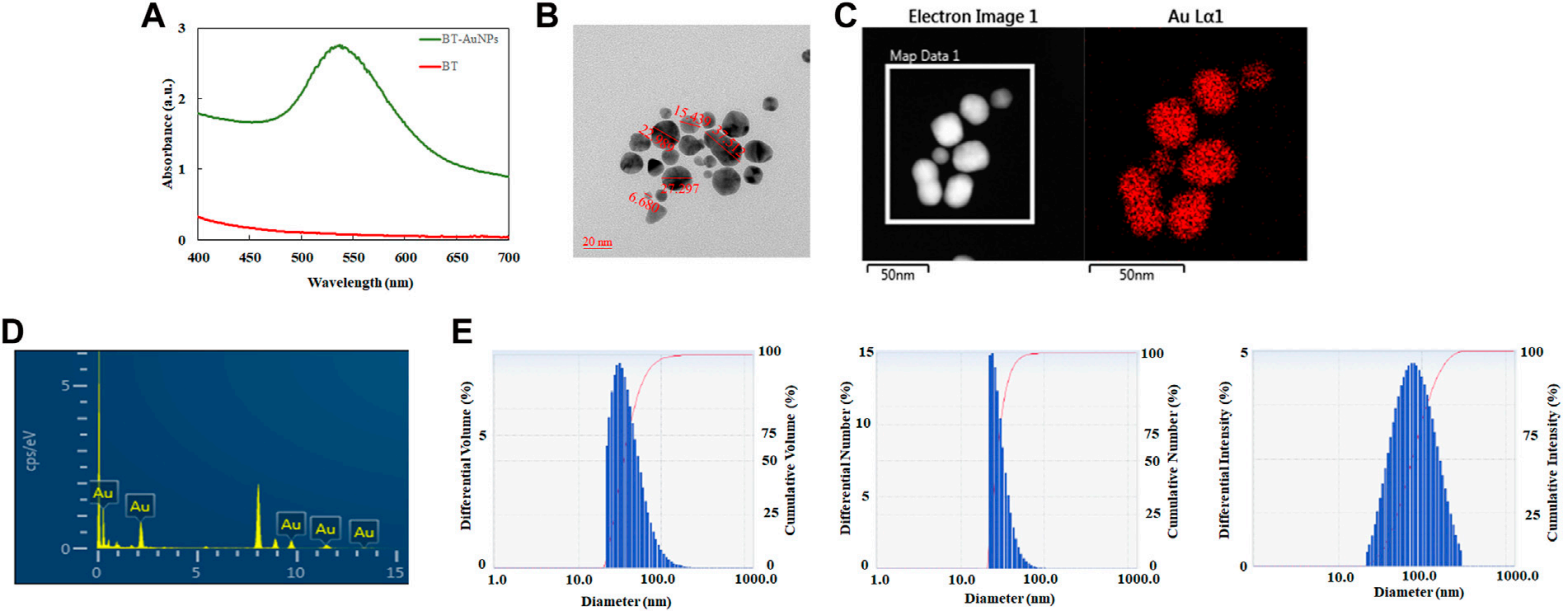
Characteration of BT-AuNPs. **(A)** UV−vis spectrum of BT-AuNPs and BT; **(B)** FE-TEM image was utilized to determine the morphology and size of BT-AuNPs. **(C)** Elemental mapping was used to confirm the gold distribution in nanoparticles; **(D)** EDX was analyzed to detect the Au peak of BT-AuNPs; **(E)** The volume, number, and intesity distributions of BT-AuNPs were determined using DLS spectrum.

BT-AuNPs were evaluated using a UV-Vis spectrophotometer. BT-AuNPs revealed a strong peak at 538 nm, corresponding to the surface plasmon band of the synthesized AuNPs (Figure 2A). Additionally, the visible color changed from yellow to purple. These results confirmed the successful synthesis of BT-AuNPs (Liu et al., 2019). Figure 2B displays the FE-TEM image of BT-AuNPs, which reveals that the particles have a size range of 6–37 nm and exhibit spherical, triangular, and polygonal surface morphologies. Most surface morphologies consisted of circular nanohybrids with a few triangular and polygonal shapes. The elemental map in Figure 2C shows the distribution of Au (red) in the isolated particles. The Energy-dispersive X-ray (EDX) spectra showed multiple absorption peaks corresponding to the characteristic Au peaks (Figure 2D). Using DLS analysis, we determined the particle profiles of BT-AuNPs. Figure 2E shows the average values for the volume, number, and intensity of the BT-AuNPs, which were 43.3, 30.5, and 95.6 nm, respectively. Furthermore, BT-AuNPs had a hydrodynamic diameter of 62.7 nm and a polydispersity index of 0.29 (<0.3), indicating a narrow size distribution of AuNPs (Ricci-Júnior and Marchetti, 2006).

**FIGURE 3:**
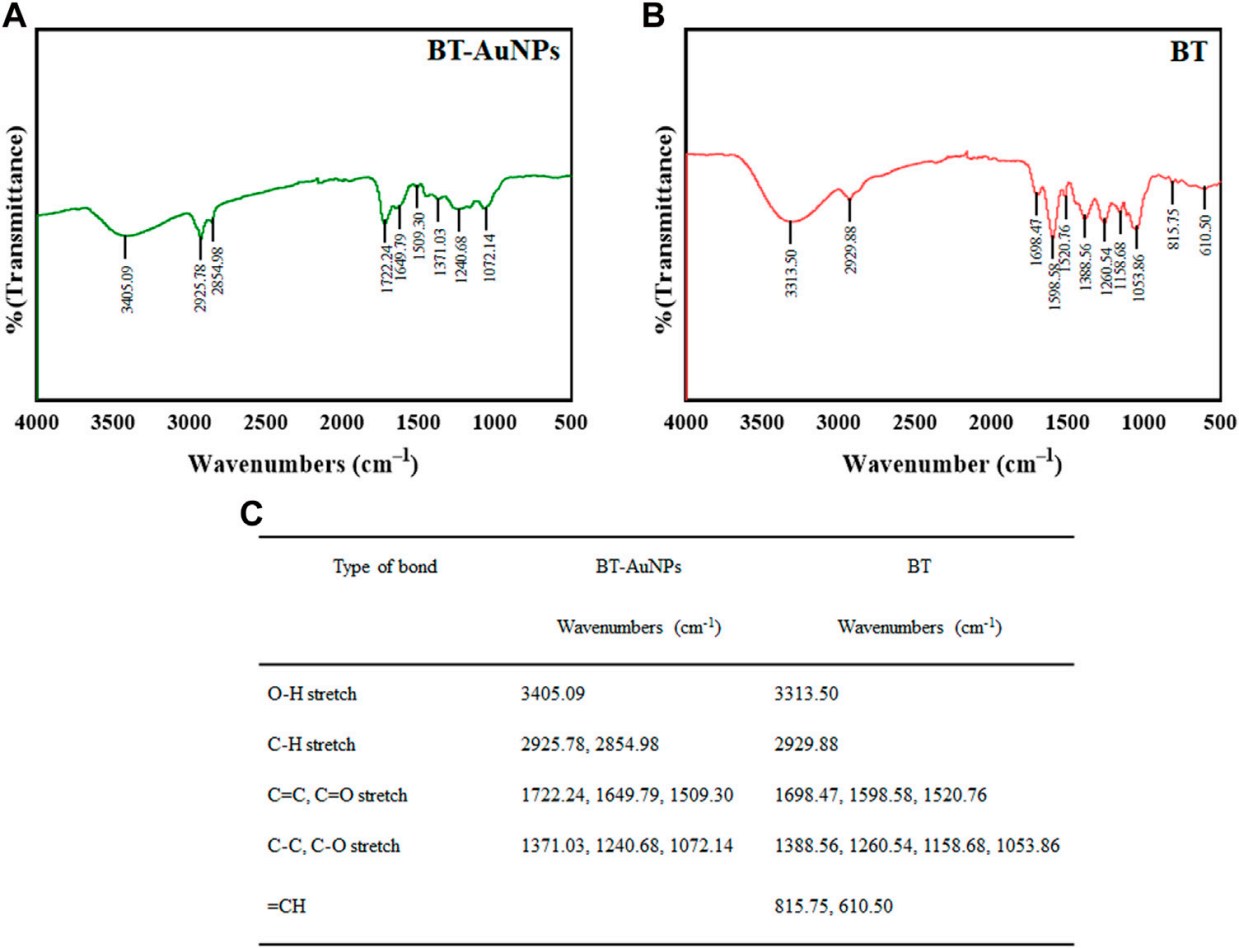
FT-IR analysis was utilized to confirm chemical linkage confirmation in BT-AuNPs. **(A)** BT-AuNPs; **(B)** BT; **(C)** The profile of a tabular view in the functional group.

The possible functional groups on the surfaces of the BT-AuNPs were identified using Fourier transform infrared (FT-IR) spectroscopy (Figures 3A, B), and the absorption peaks are shown in Figure 3C. The FT-IR spectra of BT-AuNPs and BT revealed bands at 3,405.09 cm^−1^ and 3,313.50 cm^−1^ corresponding to the O–H stretching; bands at 2,925.78–2,854.98 cm^−1^ and 2,929.88 cm^−1^ corresponding to the C-H stretching; and bands at 1,722.24–1,509.30 cm^−1^ and 1,698.47–1,520.76 cm^−1^ corresponding to the C=C and C=O stretching, respectively. The stretching of C-C-C and C-O double-bond functional groups was observed at 1,371.03–1,072.14 cm^−1^ and 1,388.56–1,053.86, respectively. The corresponding C-H band observed at 815.75–610.50 cm^−1^ was only present in BT. Therefore, we believe that HAuCl_4_⋅3H_2_O and BT combined individually to form BT-AuNPs (Mi et al., 2022a).

### 3.3 Cytotoxicity of BT-AuNPs and BT

**FIGURE 4:**
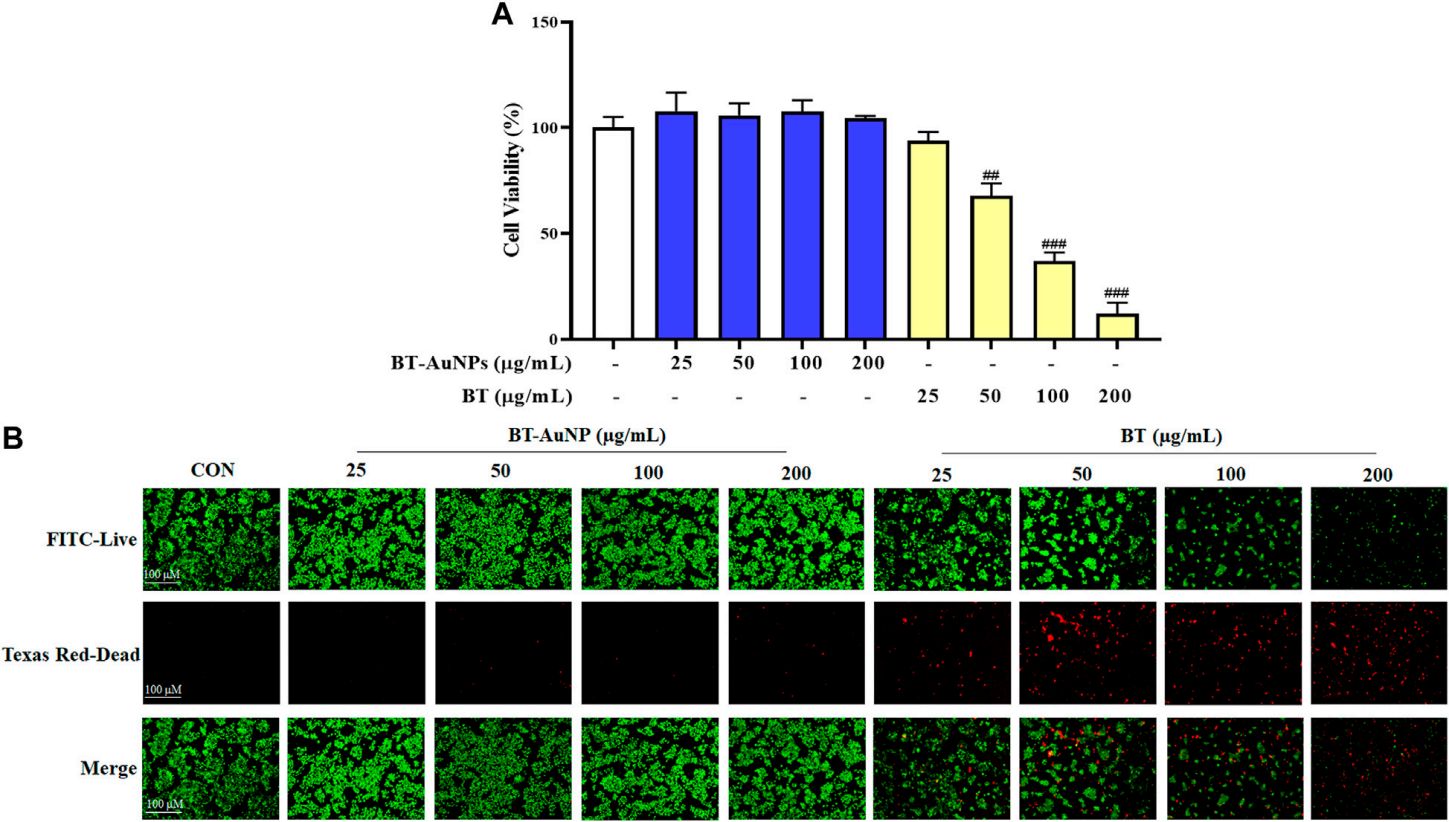
Cytotoxic of BT-AuNPs in keratinocytes. **(A)** Effects of BT-AuNPs and BT on viability in HaCaT cells were determined by MTT assay; **(B)** Live/Dead staining images were observed to confirm the cytotoxicity effect of BT-AuNPs and BT treated HaCaT cells. All values are expressed as mean ± S.D. ^##^ *p* < 0.01, ^###^ *p* < 0.001 vs. control group.

The MTT assay and live/dead cell staining were used to assess the cytotoxicity of BT-AuNPs and BT on HaCaT cells. The cells were incubated with different concentrations (25, 50, 100, and 200 μg/mL) of BT-AuNPs and BT for 24 h (Figure 4A). No significant cellular toxicity was observed on treatment with BT-AuNPs at concentrations ranging from 25 to 200 μg/mL. However, at concentrations of 50–100 g/mL, BT significantly decreased cell viability. Additionally, dead/live staining was used to determine the cytotoxic effects of BT-AuNPs and BT (Figure 4B). The data indicated that a large number of cells died in the BT-treated group, while the BT-AuNPs-treated group had relatively few dead cells. Therefore, BT-AuNPs at concentrations of 50, 100, and 200 μg/mL were chosen for further experiments.

### 3.4 Effects of BT-AuNPs on TNF-α/IFN-γ-induced pro-inflammatory cytokines/chemokines

**FIGURE 5:**
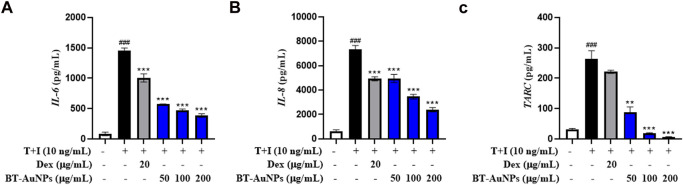
Effect of BT-AuNPs on cytokine and chemokines secretion in T + I-induced HaCaT cells were measured using ELISA assay. **(A)** *IL-6*; **(B)** *IL-8*; **(C)** *TARC*. All values are expressed as mean ± S.D. **p < 0.01, ***p < 0.001 vs*.* T + I group, ^###^ *p* < 0.001 vs*.* control group.

Chemokines and cytokines play critical roles in the immune and inflammatory responses. It is well-known that TNF-α/IFN-γ (T + I) can induce the aberrant expression of chemokines (such as IL-8, TARC, CTACK, and RANTES) and cytokines (IL-6), resulting in the infiltration of T cells or leukocytes into inflamed skin lesions (Homey et al., 2006; Nedoszytko et al., 2014). Therefore, we investigated the inhibitory effect of BT-AuNPs on pro-inflammatory chemokine and cytokine secretions induced by T + I in HaCaT cells using ELISA, with dexamethasone (Dex) as a positive control. T + I dramatically elevated the production of IL-6, IL-8, and TARC (Figures 5A–C) in keratinocytes. These increases were markedly reduced by BT-AuNPs treatment in a dose-dependent manner. Our results suggest that BT-AuNPs are promising therapeutic candidates for treating inflammatory skin disorders by inhibiting the secretion of pro-inflammatory chemokines and cytokines.

**FIGURE 6:**
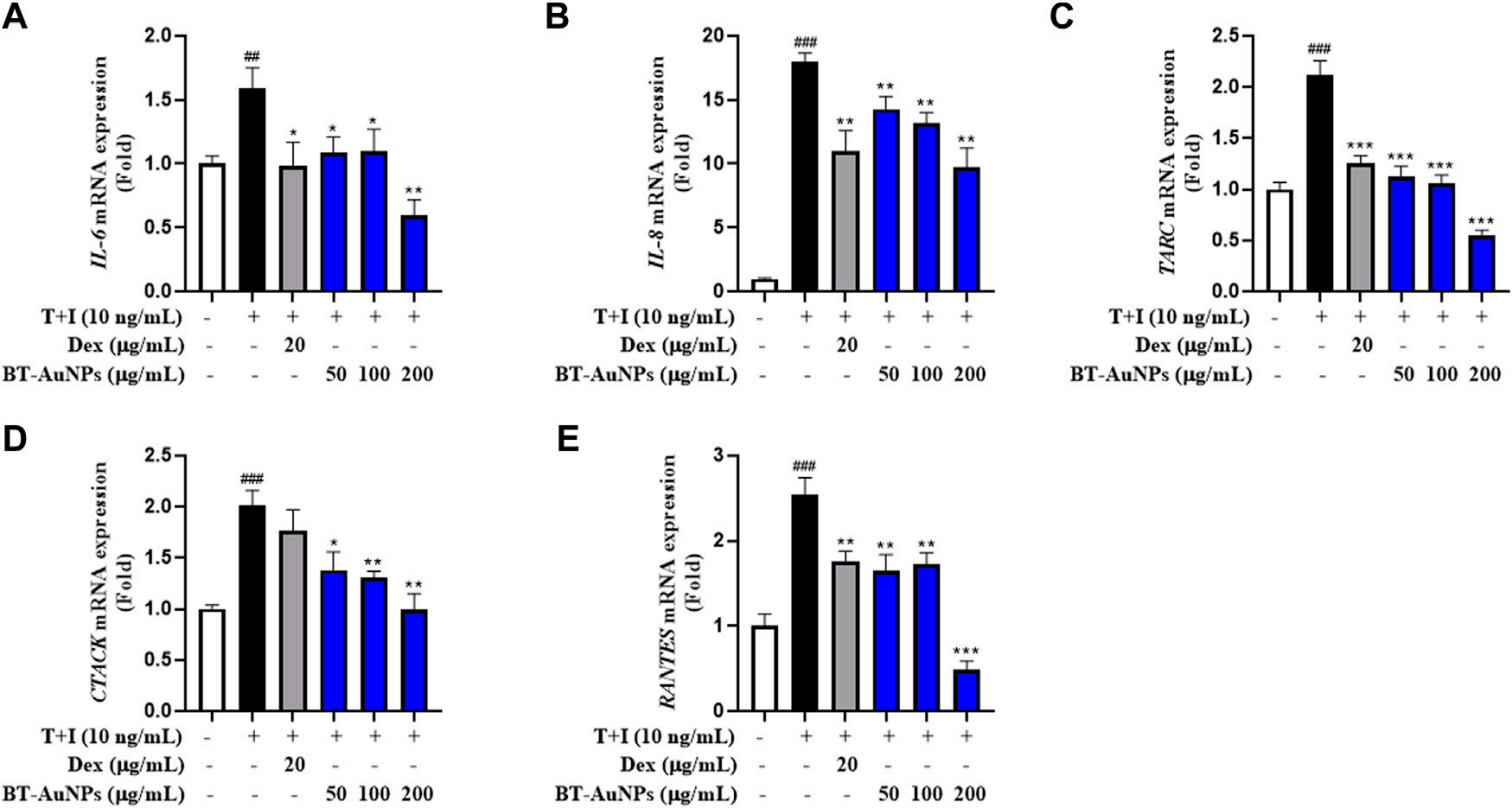
Effects of BT-AuNPs on mRNA expression in T + I-induced HaCaT cells were examined using qRT-PCR assay. **(A)** *IL-6*; **(B)** *IL-8*; **(C)** *TARC*; **(D)** *CTACK*; **(E)** *RANTES*. All values are expressed as mean ± S.D. *p < 0.05, **p < 0.01, ***p < 0.001 vs*.* T + I group, ^###^ *p* < 0.001 vs*.* control group.

Further, the mRNA expression of pro-inflammatory factors was investigated using qRT-PCR., The mRNA expression of *IL-6*, *IL-8*, *CTACK*, *TARC*, and *RANTES* (Figures 6A–E) significantly improved following T + I induction compared to those in the non-treated group. In contrast, Dex significantly suppressed the mRNA expression of pro-inflammatory chemokines and cytokines. In the group pretreated with BT-AuNPs, the levels of *IL-6*, *IL-8*, *CTACK*, *TARC*, and *RANTES* substantially attenuated. Additionally, the inhibitory effect of the BT-AuNPs was greater than that of the positive control at 200 g/mL. Overall, the ELISA and qRT-PCR results demonstrated that BT-AuNPs effectively inhibited the production and mRNA expression of pro-inflammatory chemokines and cytokines in T + I-induced HaCaT cells.

### 3.5 The anti-inflammatory mechanism of BT-AuNPs on TNF-α/IFN-γ-induced HaCaT cells

**FIGURE 7:**
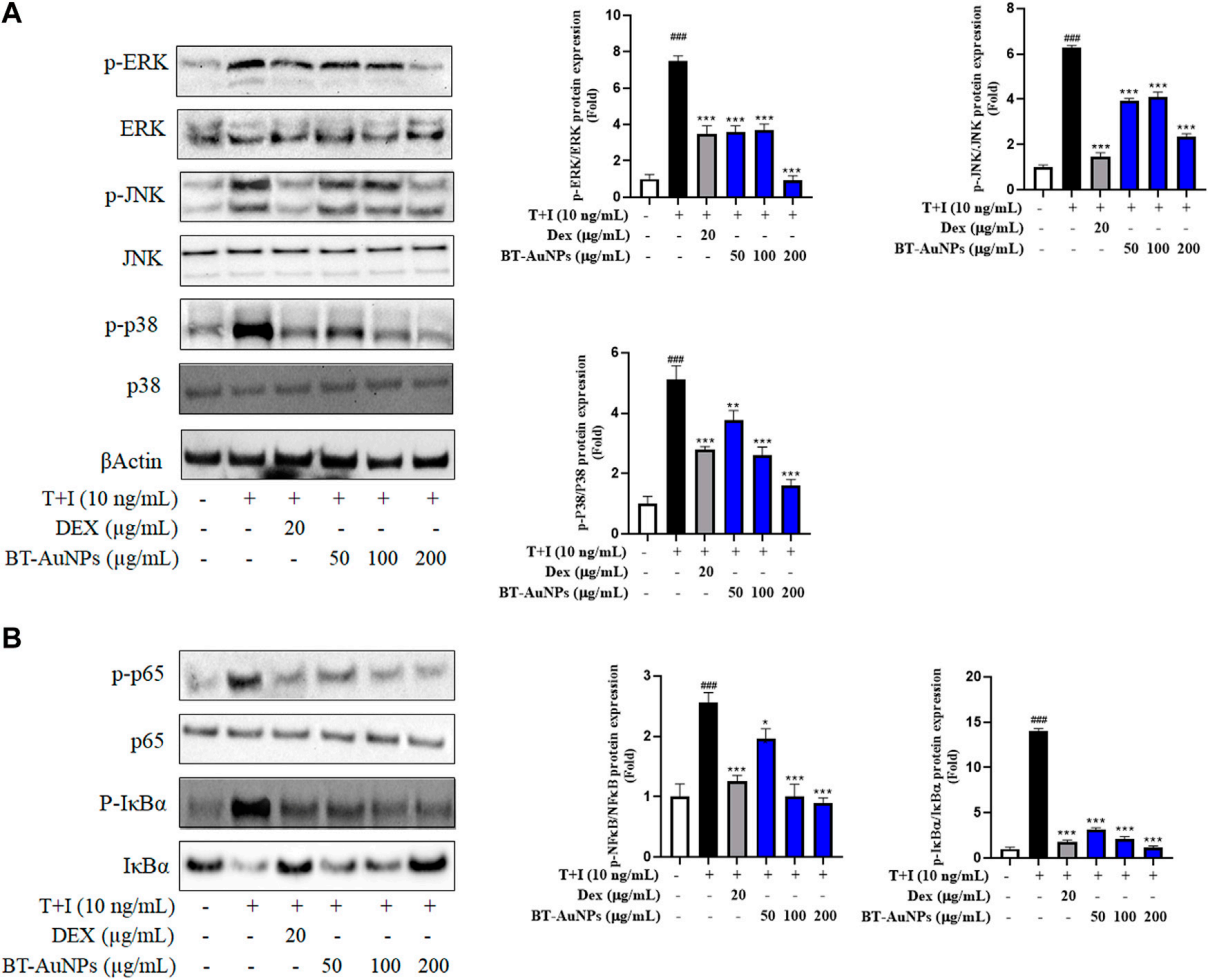
Effect of BT-AuNPs on **(A)** MAPK and **(B)** NF-κB signaling pathways in T + I-induced HaCaT cells. The protein expressions were presented as Western blotting pictures and these expressions were quantified according to the fold ratio of the phosphorylated/total form. All values are expressed as mean ± S.D. *p < 0.05, **p < 0.01, ***p < 0.001 vs*.* T + I group, ^###^ *p* < 0.001 vs*.* control group.

The MAPKs signaling pathway is crucial for various immune-mediated inflammatory responses. T + I stimulation activates the MAPKs signaling pathway, including p38, ERK, and JNK (Sikandan et al., 2018). In Figure 7A, the effects of BT-AuNPs on the activation of p38, ERK, and JNK were determined using Western blot. Immunoblotting results revealed that T + I significantly increased the protein levels of phosphorylated p38, ERK, and JNK; however, these increases were significantly attenuated by BT-AuNPs. Similarly, Dex markedly reduced the levels of phosphorylated MAPKs signaling pathway proteins. Interestingly, at the highest concentration of BT-AuNPs, the inhibitory effect on ERK and p38 phosphorylation was greater than that of the positive control. These findings imply that BT-AuNPs suppress the T + I-induced activation of the MAPKs signaling pathway.

Moreover, NF-κB can regulate the inflammatory response by producing chemokines and cytokines (Ju et al., 2009). To investigate the impact of BT-AuNPs on the NF-κB signaling pathways, we analyzed the levels of IκB-α degradation, phosphorylated IκB-α, and phosphorylated p65. As shown in Figure 7B, compared to the control, T + I treatment significantly degraded IκB-α and increased the levels of phosphorylated IκB-α and p65. However, compared to the T + I alone group, pretreatment with BT-AuNPs markedly reduced p-P65 and p-IκB-α levels. Furthermore, BT-AuNPs significantly restored IκB-α protein levels, which were degraded by T + I. Consequently, our results suggest that BT-AuNPs inhibit the phosphorylation of IκB-α and p65 and prevent T + I-stimulated IκB-α degradation.

### 3.6 Effect of BT-AuNPs on hyaluronic acid production and related gene and protein expression

**FIGURE 8:**
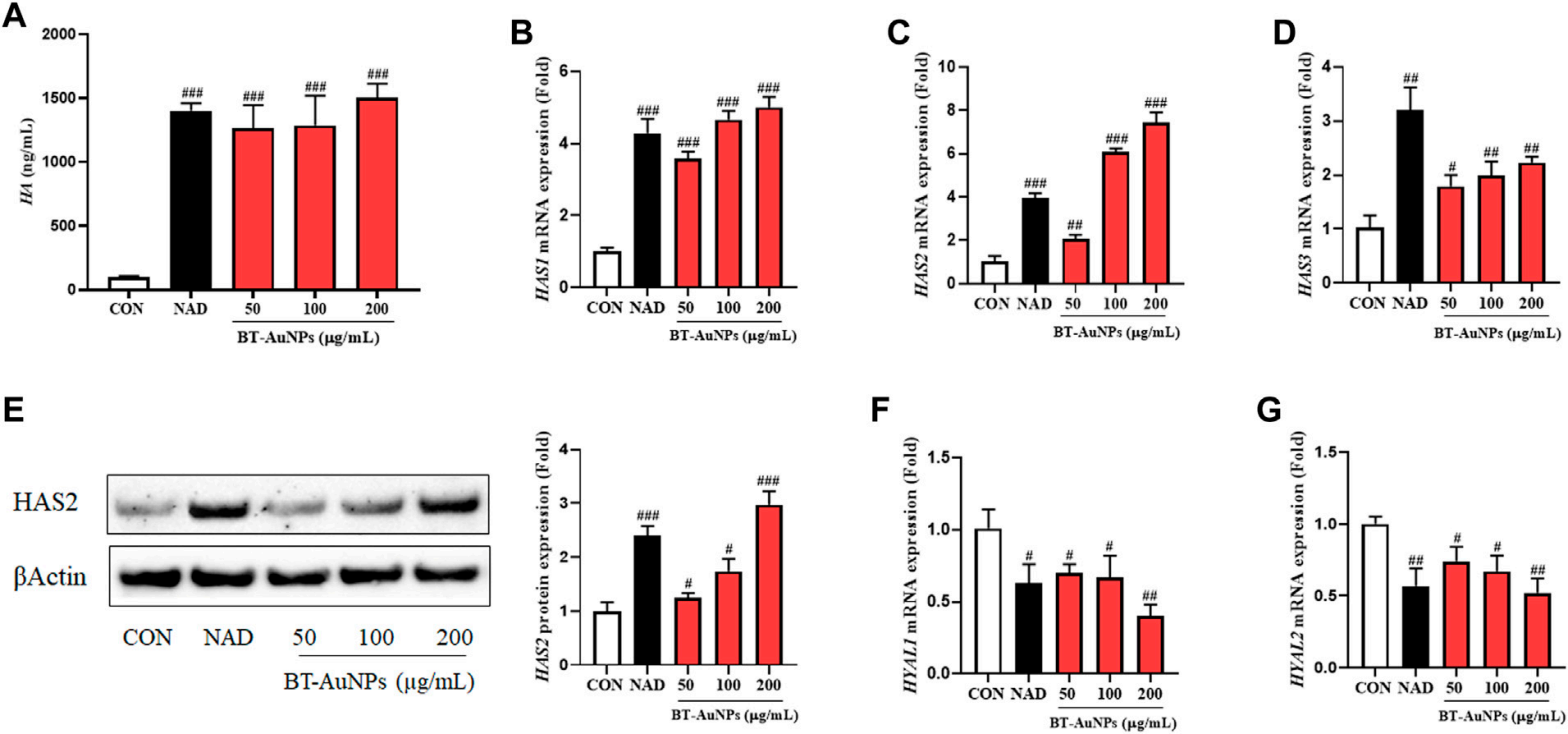
Effect of BT-AuNPs on HA production in normal keratinocytes. **(A)** HA secretion; gene expression of **(B)** *HAS1*, **(C)** *HAS2*, **(D)** *HAS3*; protein expression of **(E)** HAS2; gene expression of **(F)** *HYAL1*, **(G)** *HYAL2*. ELISA assay was conducted to measure HA secretion, and qRT-PCR was used to measure the expression of HA-related mRNA. All values are expressed as mean ± S.D. ^##^ *p* < 0.05, ^##^ *p* < 0.01, ^###^ *p* < 0.001 vs*.* control group.

Moisture maintenance is essential for normal skin function and is critical in regulating physiological processes in the skin, such as inflammation and wound recovery (Lee et al., 2019; Dias et al., 2021; Mi et al., 2022c). Thus, the effects of BT-AuNPs on skin moisture were evaluated in normal HaCaT cells using ELISA, Western blotting, and qRT-PCR. *N*-acetyl-D-glucosamine (NAD) was used as a positive control. HA production significantly improved in a dose-dependent manner in the BT-AuNP-treated group compared to that in the untreated group (Figure 8A). Notably, 200 μg/mL BT-AuNPs were more effectively produced HA than that by the NAD group. Moreover, BT-AuNPs enhanced the mRNA expression of HA synthesis (HAS) genes, such as *HAS1*, *HAS2*, and *HAS3* (Figures 8B–D). Interestingly, HAS2 mRNA expression levels in the BT-AuNP-treated group at concentrations of 100 (6.1 fold) and 200 (7.4 fold) µg/mL were higher than that in the NAD (3.9 fold) group. Controlling HAS2 expression can preserve homeostasis and moisture in keratinocytes (Mi X. et al., 2022c). Thus, we further confirmed the moisturizing effect of BT-AuNPs on the HAS2 protein using Western blotting. The protein expression of HAS2 was dose-dependently enhanced by BT-AuNPs (Figure 8E) compared with that in the normal control. Additionally, the mRNA expression of hyaluronidases (*HYALs*, *HYAL1*, and *HYAL2*) was strongly suppressed after BT-AuNPs treatment (Figures 8F,G). These data indicate that BT-AuNPs may promote the production of HA by increasing the expression of HA-synthesizing enzymes and inhibiting HA-degrading enzymes.

### 3.7 Identification of phytochemicals for BT-AuNPs synthesis

The major active compounds in BT were determined using a UPLC-MS/MS analysis. PDA (Photodiode array chromatogram) and BPC (base peak chromatogram) of BT are exhibited in Supplementary Figure S1. To calculate mass-to-charge ratio (m/z) and identify major peaks with mass accuracy less than ±5 ppm, mass spectrometry (MS) and tandem MS (MS^2^) were used. On the BPC, two peaks at retention periods of 6.49 and 6.70 min were verified to correspond to their source ions at 611.1581 and 465.1012, respectively, according to positive ionized mode [(M + H)] +) MS analysis. Thus, the results of MS and MS^2^ indicated that BT may contain 2 major phytochemicals, such as rutin and hyperoside (quercetin-3-O-galactoside).

## 4 Discussion

AuNPs are widely utilized in biomedical applications to treat various diseases, including inflammatory diseases, cancer, and immune system disorders (Xu et al., 2021; Mi et al., 2022b; Mi et al., 2022a). In this study, we synthesized BT-AuNPs using BT extract as a green reducing agent and investigated their bioactivity. Previous study showed that once the AuNPs are synthesized, the visual color of the reaction mixture changes to a deep purple or ruby red due to the surface plasmon resonance (Ahn et al., 2018). The color change of the reaction mixture from light yellow to dark purple, clearly suggesting that the BT extract is capable of reducing HAuCl_4_⋅3H_2_O to synthesize AuNPs. Next, analysis of the synthesis conditions of the nanoparticles indicated that BT-AuNPs were successfully synthesized under optimized conditions. The UV-vis absorption spectra of the reaction mixtures showed that there was a appearance of BT-AuNPs peak at λmax = 538 nm, which corresponds to the characteristic surface plasmon resonance phenomena of AuNPs. Further, the characteristics (crystalline nature, particle shape, and size) of the BT-AuNPs were determined using UV-Vis spectroscopy, FE-TEM, EDX, DLS, and FTIR analyses. FE-TEM exhibited particle sizes ranging from 6 to 37 nm and structures with almost spherical, a few triangular, and polygonal shapes, similar to those observed in a previous study (Salunke et al., 2014). The presence of typical metallic gold nanocrystal structure patterns was also confirmed using EDX analysis. Since DLS analysis showed that the size of the nanoparticles was dependent on the total size of the conjugates or their hydrodynamic size in colloids, the sizes obtained were normally considerably larger than those acquired using FE-TEM analysis (Colacio et al., 1996). AuNPs that are hydrodynamically smaller than 100 nm may be phagocytosed and internalized by caveolae and clathrin, indicating BT-AuNPs may have high biocompatibility (Colacio et al., 1996). The FT-IR spectra of the BT-AuNPs showed that the hydroxyl groups of phenolic compounds may contribute to capping and bioreduction during the synthesis of AuNPs (Ahn et al., 2018).

MTT assay and live/dead staining results suggested that BT-AuNPs were non-toxic to cells at concentrations ranging from 25 to 200 μg/mL, whereas BT was highly toxic to epidermal keratinocytes at concentrations 100 and 200 μg/mL. Similar to our results, a previous study has reported that *Rosa rugosa*-based Au nanoparticles are safer than the *R. rugosa* extract, indicating that the synthesis of BT-AuNPs may reduce the cytotoxicity of BT (Wang et al., 2022a). Further, we determined the anti-inflammatory properties of BT-AuNPs on T + I-stimulated HaCaT cells. Keratinocytes functionally constitute the epidermal barrier and play an important role in the pathophysiology of inflamed skin (Jiang et al., 2020). When keratinocytes are damaged by various stimuli, chemokines and cytokines are produced that stimulate inflammatory skin diseases (Tsai et al., 2022). Subsequently, immune cells, including neutrophils, macrophages, and T cells, are also stimulated and respond to inflammatory skin lesions (Wang et al., 2022b). The immune cells recruited to inflammatory skin lesions secrete chemokines such as IL-8, TARC, RANTES, and CTACK, as well as cytokines such as IL-4 and IL-6 (Si-Si et al., 2011; Mi et al., 2022c; Tsai et al., 2022). Accordingly, various studies have used T + I-stimulated HaCaT cell models to investigate their *in vitro* potential as skin anti-inflammatory agents (Albanesi and Pastore, 2010; Lim et al., 2015; Yang et al., 2018). Herein, T + I was used as an inflammation-inducing agent to investigate the anti-inflammatory properties of BT-AuNPs on keratinocytes. BT-AuNPs considerably decreased the mRNA expression of T + I-stimulated inflammatory genes, such as *IL-6*, *IL-8*, *CTACK*, *TARC*, and *RANTES*. Furthermore, the levels of *IL-6*, *IL-8*, and *TARC* decreased considerably after BT-AuNPs pretreatment. Similar to our results, AuNPs synthesized using *R. rugosa* extracts also inhibit inflammation in T + I-stimulated keratinocytes cells by suppressing chemokine/cytokine secretion and production (Wang et al., 2022a). These results suggested that BT-AuNPs could effectively attenuate inflammation in T + I-induced keratinocytes by inhibiting inflammatory cytokines and chemokines, such as IL-6, IL-8, CTACK, TARC, and RANTES.

Moreover, T + I can activate numerous intracellular signaling pathways, such as NF-κB and MAPK pathways, which are associated with inflammatory diseases like atopic dermatitis (Basu et al., 2019; Cho et al., 2020). The keratinocytes in patients with atopic dermatitis secrete chemokines and cytokines that stimulate the development of ISDs, which is triggered by MAPK signaling (Sung et al., 2012; Lim et al., 2016). The protein expression of phosphorylated ERK, JNK, and p38 were dramatically reduced in T + I-induced keratinocytes after BT-AuNPs pretreatment, implying that BT-AuNPs inhibit T + I-stimulated MAPK pathway activation. The MAPK signaling is important in immune responses and collaborates with NF-κB to modulate inflammatory pathways (Kim et al., 2010; Sung et al., 2012). Our results indicated that BT-AuNPs also markedly suppressed the protein expression of phosphorylated IκB-α and p65, suggesting that BT-AuNPs might inhibit T + I-stimulated NF-κB signaling activation which includes release and phosphorylation of IκB, NF-κB translocation into the nucleus, and activation of the inflammatory response (Mi et al., 2022c). Activation of MAPK and NF-κB pathways releases pro-inflammatory cytokines and chemokines, such as *IL-6* and *IL-8,* potentially leading to an inflammatory response (Tang et al., 2017; Xiao et al., 2020). NF-κB and p38 are partially responsible in regulating the mechanism for *TARC* production in keratinocytes (Qi et al., 2009). Thus, BT-AuNPs inhibited the production of inflammation chemokines and cytokine via inhibiting the activation of NF-κB and MAPK pathways in keratinocytes.

HA is an important extracellular glycosaminoglycan component, which is associated with physiological processes of the skin like anti-aging, skin moisturizing, anti-inflammation, wound recovery, skin repair, and tissue regeneration (Bukhari et al., 2018; How et al., 2020). HA is a potential drug delivery agent in ISDs, such as psoriasis and atopic dermatitis (How et al., 2020; Marinho et al., 2021). Using HA or identifying substances that promote HA manufacturing are potential strategies to maintain skin health and cure a variety of skin-associated disorders (Qu et al., 2014; Mi et al., 2022c). HA is produced by the catalytic action of HAS, including *HAS1*, *HAS2*, ad *HAS3* and conversely its depletion is promoted by hyaluronidase, including *HYAL1* and *HYAL2* (Oh et al., 2022). Therefore, the effect of BT-AuNPs on HA secretion was further examined via the analysis of related signaling molecules in HaCaT cells. ELISA and qRT-PCR results showed that BT-AuNPs remarkably increased HA secretion and HASs family (*HAS1*, *HAS2*, *HAS3*) mRNA expression. Notably, at a concentration 200 μg/mL BT-AuNPs, the gene expressions of *HAS1* (5.0 fold) and *HAS3* (2.2 fold) were lower than that of *HAS2* (7.4 fold). Among the HAS family members, HAS2 is mainly expressed in organisms and plays a key role in HA production (Kim et al., 2021). Thus, the protein expression of HAS2 was investigated using Western blot analysis. As expected, BT-AuNPs increased the expression of the HAS2 protein in a concentration-dependent manner, implying that HAS2 might play a crucial role in HA secretion in keratinocytes following BT-AuNPs treatment. Moreover, BT-AuNPs significantly downregulated the expression of *HYAL1* and *HYAL2* in HaCaT cells. Consistent with our results, AuNPs synthesized using *Diospyros kaki* fruit extracts also stimulate the *HAS* genes and inhibit *HYAL*s, leading to improved HA secretion (Dhandapani et al., 2023). In summary, the above results suggest that BT-AuNPs may stimulate HA production by promoting HA synthesis (*HAS1*, *HAS2*, and *HAS3*) and suppressing hyaluronidase (*HYAL1* and *HYAL2*).

Finally, we determined the compounds that are critical to the anti-inflammatory and moisturizing effects of BT. Two major flavonoids, including rutin and hyperoside (quercetin-3-O-galactoside), were identified as the primary phytochemicals in BT by UPLC-MS/MS analysis. Similar with our findings, a previous study identified two flavonoids (rutin and isoquercetin) from *B. tricuspis* extract using HPLC analysis (Akter et al., 2018). Notably, both rutin and hyperoside were reported to have anti-inflammation properties on UVB irradiated keratinocytes (Gęgotek et al., 2019; Charachit et al., 2022). Kamel and Mostafa (2015) suggested that nanocream prepared from rutin exhibit moisture-enhancing properties for the skin. Therefore, these flavonoids may contribute to the an anti-inflammatory activity or HA production of BT-AuNPs.

## 5 Conclusion

In this study, we successfully synthesized uniformly shaped BT-AuNPs using a combination of the BT extract and Au salts. Our findings highlight the non-toxic effects of BT-AuNPs, whereas BT exhibits significant toxicity in HaCaT cells at the same concentration. This implies that the green-synthesized NPs can effectively reduce the toxicity of BT in HaCaT cells. Furthermore, BT-AuNPs greatly suppressed the inflammatory response via inhibiting NF-κB and MAPK pathways in T + I-stimulated keratinocytes. Additionally, we found that BT-AuNPs stimulated HA production by regulating the expression of HAS and HYAL biomarkers. Thus, our results provide valuable evidence of the anti-inflammatory and moisturizing properties of green-synthesized AuNPs. Therefore, BT-AuNPs can potentially be used as effective agents for treating inflammatory skin diseases and promoting skin health.

## Data Availability

The original contributions presented in the study are included in the article/Supplementary Material, further inquiries can be directed to the corresponding authors.
