# Supplementary material for: The anti-inflammation and skin-moisturizing effects of Boehmeria tricuspis-mediated biosynthesized gold nanoparticles in human keratinocytes
Source: Front Pharmacol. 2023 Oct 6;14:1258057. doi: 10.3389/fphar.2023.1258057 (PMC10588637; doi:10.3389/fphar.2023.1258057)
Supplement: Supplementary file 1 [file DataSheet1.DOCX]

**The Anti-Inflammation and Skin-Moisturizing Effects of *Boehmeria tricuspis*-mediated Biosynthesized Gold Nanoparticles in Human Keratinocytes**

Thi Hoa My Tran^1^, Rongbo Wang^1^, Hoon Kim^2*^ and Yeon-Ju Kim^1*^

^1^Graduate School of Biotechnology, and College of Life Science, Kyung Hee University, Deogyeong-daero 1732, Giheung-gu, Yongin 17104, Republic of Korea

^1^Department of Food and Nutrition, Chung Ang University, Seodong-daero 4726, Daedeok-myeon, Anseong 17546, Republic of Korea

* Correspondences:

Yeon-Ju Kim, Email: [yeonjukim@khu.ac.kr](mailto:yeonjukim@khu.ac.kr), Tel: +82-31-201-2645; Graduate School of Biotechnology, and College of Life Science, Kyung Hee University, Deogyeong-daero 1732, Giheung-gu, Yongin 17104, Republic of Korea

Hoon Kim, Email: [saphead1106@hanmail.net](mailto:saphead1106@hanmail.net), Tel: +82-31-888-6180; Department of Food and Nutrition, Chung Ang University, Seodong-daero 4726, Daedeok-myeon, Anseong 17546, Republic of Korea


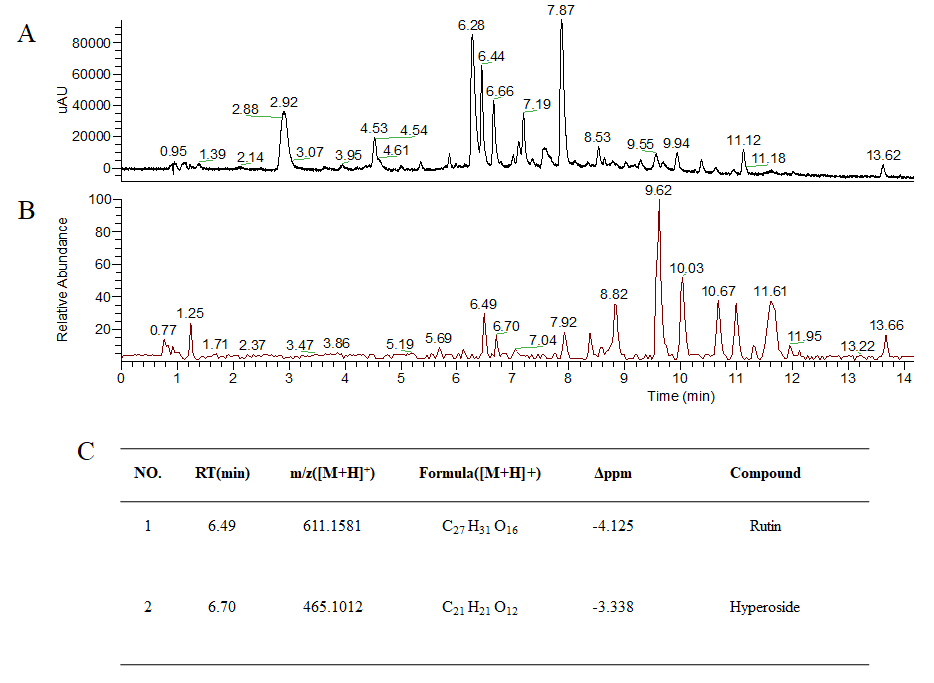


Figure S1.

A. Photo-diode array chromatogram (PDA), B. base peak chromatogram (BPC) of BT obtained by UPLC-MS analysis, C. The compound identification of two major phytochemicals in BT.

Table S1. Primer sequences used for qRT–PCR analysis in this study

| Primer name | Forward  Reverse | Sequence (5′→3′) |
| --- | --- | --- |
| IL-8 | Forward  Reverse | 5′–GAGAGTGATTGAGAGTGGACCAC–3′  5′–CACAACCCTCTGCACCCAGTTT–3′ |
| TARC | Forward  Reverse | 5′–TGTAAAACGACGGCCAGT–3′  5′–CAGGAAACAGCTATGACC–3′ |
| RANTES | Forward  Reverse | 5′–CATATTCCTCGGACACCACACCCT–3′  5′–ACTCCTGACCTCAAGTGATCCACC–3′ |
| CTACK | Forward  Reverse | 5′–CACTGCCTGCTGTACTCAGCTCTA–3′  5′–CTTCAGCCCATTTTCCTTAGCATC–3′ |
| HAS1 | Forward  Reverse | 5′–CCTCACCAACCGCATGCT–3′  5′–GGACGAGGGCGTCTCTGA–3′ |
| HAS2 | Forward  Reverse | 5′–CTGGGACGAAGTGTGGATTATG–3′  5′–GATGAGGCTGGGTCAAGCAT–3′ |
| HAS3 | Forward  Reverse | 5′–GCCCTCGGCGATTCG–3′  5′–TGGATCCAGCACAGTGTCAGA–3′ |
| HYAL1 | Forward  Reverse | 5′–CCTCACCAACCGCATGCT–3′  5′–TCCTTGATGGCCTGACATGA–3′ |
| HYAL2 | Forward  Reverse | 5′–GCACTCCCAGTCTACGTCTTCA–3′  5′–GCACTCTCGCCAATGGTAGAG–3′ |
| GAPDH | Forward  Reverse | 5′–ACCACAGTCCATGCCATCAC–3′  5′–CCACCACCCTGTTGCTGTAG–3′ |
